# A New Treatment for Mammillary Fistulas Using Ultrasound-Guided Percutaneous Needle Electrolysis

**DOI:** 10.3390/jcm9030649

**Published:** 2020-02-28

**Authors:** Juan de Dios Berná-Serna, José A. García-Vidal, María P. Escolar-Reina, Francesc Medina-Mirapeix, Florentina Guzmán-Aroca, Antonio Piñero-Madrona, Juan de Dios Berná-Mestre

**Affiliations:** 1 Department of Radiology, Virgen de la Arrixaca University Clinical Hospital, Ctra. Madrid-Cartagena, El Palmar, 30120 Murcia, Spain; jdberna@gmail.com (J.d.D.B.-S.); masjubermu@hotmail.com (J.d.D.B.-M.); 2 Institute of Biomedical Research (IMIB)-Virgen de la Arrixaca University Clinical Hospital, El Palmar, 30120 Murcia, Spain; garciavidal@um.es (J.A.G.-V.); pescolar@um.es (M.P.E.-R.); mirapeix@um.es (F.M.-M.); pineromadrona@gmail.com (A.P.-M.); 3 Departament of Physiotherapy, University of Murcia, Campus de Espinardo, 30100 Murcia, Spain; 4 Department of Surgery, Hospital Clínico Universitario “Virgen de la Arrixaca” and University of Murcia, 30120 Murcia, Spain

**Keywords:** breast, mammillary fistulas, percutaneous needle electrolysis, ultrasound

## Abstract

The aim of this study was to investigate the efficacy of ultrasound-guided percutaneous needle electrolysis (PNE) in mammillary fistulas (MFs). A prospective study was performed in 18 patients with MF who were treated with the PNE technique. The technique was repeated in the case of no response or recurrence. The results obtained show that MFs revealing an elongated appearance with the ultrasound (US) are generally resolved with two sessions of PNE, whereas ovoid MFs require several sessions of PNE for complete resolution and they tend to recur. Success of the treatment with PNE was observed in 88.8% of the patients (16/18), and failure, after five or six sessions in two cases (11.2%), which were referred for surgery. Conclusions: To the best of our knowledge this is the first study to reveal that the PNE technique is safe, effective, quick, and well-tolerated by patients.

## 1. Introduction

Mammary fistula (MF) is commonly associated with a non-lactating subareolar abscess and affects young women [1,2,3,4]. The subareolar abscess can drain spontaneously around the areola, forming a track that can usually lead to a chronic sinus or fistula. The disease can cause prolonged morbidity and has a profound impact on the quality of life. The main problems are recurrence [3,5] and the resulting reoperations. The best management of MFs is not standardized and remains a great challenge.

The cause of MFs is still unknown. This entity is commonly considered to be closely linked to squamous metaplasia with keratinization and/or epidermalization of the lactiferous duct [6,7]. However, it is also postulated that MFs might be due to a chronic inflammatory process of the pilosebaceous follicles in a periareolar location [8]. On the basis of this new theory, we consider percutaneous needle electrolysis (PNE) as a useful tool in the treatment of MF [9].

PNE is a technique that involves the application of a galvanic current through an ultrasound (US)-guided needle that produces a non-thermal electrochemical ablation using a cathode flow (the needle acts as a negative pole or cathode) directly into the affected tissue [10]. This process results in the formation of molecules of sodium hydroxide, which causes the destruction of the damaged tissue, producing localized inflammation—exclusively in the treatment zone—leading to the rapid regeneration of such tissue [10,11,12,13]. This technique has been proved efficient in tendinopathies [10,11,12].

To our knowledge, this is the first study to describe PNE as a technique for the treatment of MFs. The aim of this study, therefore, was to investigate the efficacy of this technique in the management of these MFs.

## 2. Material and Methods

### 2.1. Patients

This prospective study was conducted according to the guidelines of the Helsinki Declaration and written approval was obtained by our Institutional Ethics Committee (“Virgen de la Arrixaca” University Hospital IRB Reference number: 2018-07-04-HCUVA), to investigate the safety and effectiveness of PNE in patients with MF. Written informed consent was obtained from all the participants before their enrolment into the study. Between June 2017 and July 2019, we recruited 18 consecutive patients (17 females and 1 male) with MF. Inclusion criteria for the study were age older than 18 years, MF, and consent for recruitment by the patient. Exclusion criteria were pregnancy, oncology patient, breast prosthesis, pacemaker, endoprosthesis, and osteosynthesis. One patient declined to participate in the study.

### 2.2. Description of Procedure

**Figure 1:**
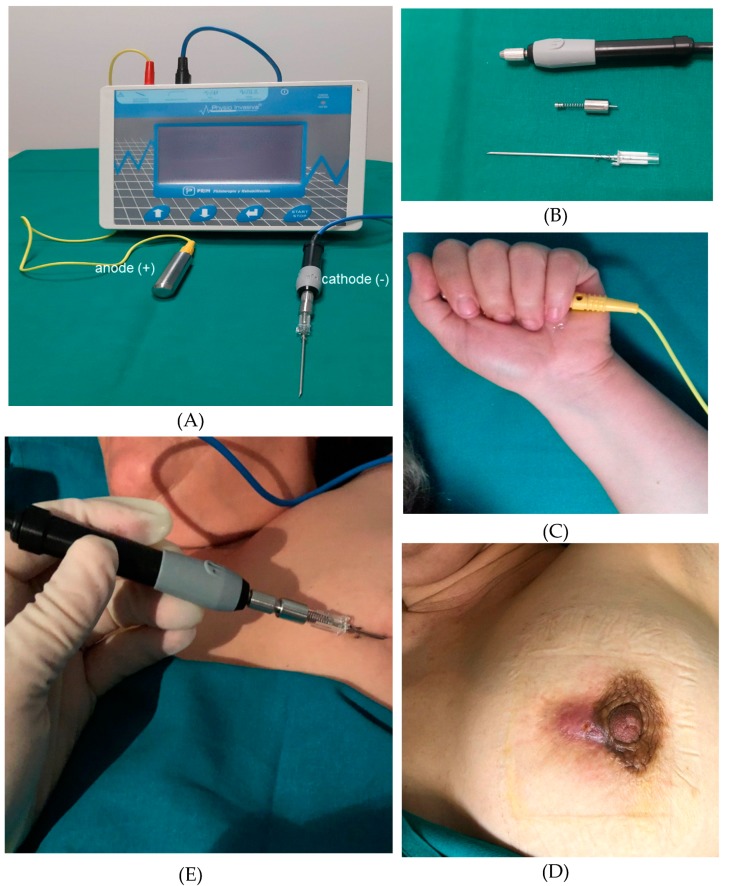
(**A**) Percutaneous needle electrolysis (PNE) unit employed in the study and electrodes; (**B**) needle holder (cathode), adapter to connect the needle to the needle holder and needle with the safety shield moved to the base of the needle; (**C**) patient holding a hand-held anode; (**D**) 46-year-old woman with a mammary fistula (MF); (**E**) PNE treatment.

**Figure 2:**
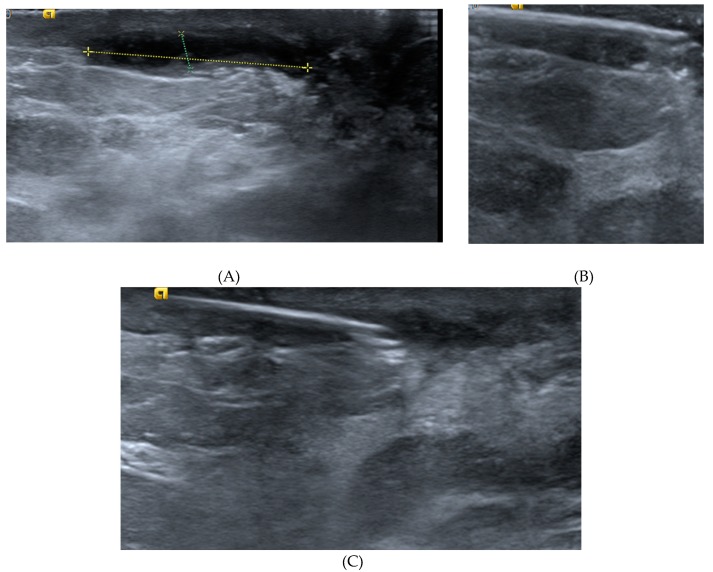
Example of PNE of an MF: (**A**) the ultrasound image shows a hypoechoic elongated appearance; (**B**) needle inserted into the track distal zone; (**C**) hyperechoic image over the needle after application of PNE.

The US-guided PNE procedure was performed by a radiologist with 29 years’ breast imaging experience using an Acuson S2000 ultrasound system (Siemens, Erlangen, Germany) equipped with an 18L6HD transducer or Philips EPIQ 7 with an 18–5L MHz (Philips Healthcare, Bothell, WA, USA); for the PNE we used a Physio Invasiva^®^ device (PRIM Fisioterapia, Madrid, Spain), which produces a continuous galvanic current through the cathode while the patient holds a hand-held anode (Figure 1). Rigorous aseptic measures were applied. The skin was cleaned with 2% chlorhexidine and local anesthetic (2% mepivacaine) was injected into the puncture site (1–2 mL). A 14G Abbocath needle was inserted into the MF track after the catheter was removed and the safety shield opened slightly to move it to the base of the needle, and fluid aspiration and lavage with sterile saline were performed. Moreover, in the cases presenting with an abscess, the cavity was aspirated and then irrigated several times with saline solution. The needle was then connected to the needle holder (cathode) using an adapter designed in the mechanic’s workshop at our university. The PNE session included the application of 5 impacts at 5 mA lasting 5 s, whilst observing the hydrogen gas produced by the electrolytic reaction as a hyperechoic image over the needle (Figure 2), which was performed by withdrawing the needle at 1 cm intervals from the most distal area of the track to the skin. Moreover, in the case of patients with a cutaneous orifice, the needle was also inserted approximately 1 cm into the orifice and another 5 impacts were applied. All the patients had previously received antibiotic therapy as recommended by their physicians and were treated with an oral antibiotic for 7–10 days; amoxicillin plus clavulanic acid was used. This treatment was suspended after the procedure, in order to isolate the effect of the PNE. In addition, according to reference authors [13], galvanic currents have a bactericidal effect. Clinical follow-up and US examinations were performed at 1 and 2 weeks, and at 1, 3, 6, and 12 months. The cost of each PNE session comprised of materials required such as needles, gloves, gauze, and local anesthetic (€10), as well as, the professional fees of the time spent on the procedure (10 min).

The response to treatment was assessed as complete response (CR) or no response (NR). A CR was considered when there were no residual symptoms and the US showed the disappearance of the anechoic image after the first session. NR was considered when there was partial disappearance of the hypoechoic tubular image and the patient continued with suppuration after the first session. In the NR patients, additional US-guided PNE was administered at 2-week intervals. Success was defined as no MF recurrence during the six months following the start of the PNE treatment, and failure was considered when a new MF recurrence was observed after 5 or 6 sessions of PNE (in patient CRs previously).

### 2.3. Data Collection

We selected 14 variables among the covariates identified in literature research showing potential associations with either remission or time to remission of MFs. The information recorded for each patient included age, duration of symptoms, height, weight, body mass index (BMI), smoking, nipple inversion, periareolar cutaneous orifices, and periareolar/subareolar abscesses. Aspiration of pus confirmed a breast abscess. Samples of the aspirates were sent for microbiological examination. The ultrasound characteristics of the fistulas (location, measurement of the cavity shape: maximum diameter and width, and ultrasound appearance) were recorded. The MFs were classified into two groups according to their US appearance: (1) Elongated, when the US transverse section shows a hypoechoic image that appears parallel to the skin; and (2) Ovoid, when both the US transverse section and the sagittal section show a hypoechoic image that appears to be saccular. 

The time of the US-guided PNE session and the number of sessions were also determined. At the end of the procedure, pain intensity was registered using a visual analog scale of 0 to 10, where 0 meant no pain, and 10 meant the worst possible pain. Likewise, the results of the PNE were recorded (CR or NR), together with the number of sessions of PNE, and months of follow-up.

### 2.4. Statistical Analysis

Descriptive statistics were used to characterize the cohort at baseline and to describe the types of responses to treatment such as the number of remissions and sessions at the end of the study period. Cox’s proportional hazards regression was used for univariate analysis of demographic, clinical and echographic variables influencing the time to the first remission without relapse, within the following six months. To explore the assumption of the proportionality of the risks we used the Log Minus Log (LML) plot. The groups defined by meaningful and categorical variables (*p* < 0.05) from the univariate analyses were compared in order to identify significant differences. Normally distributed continuous variables were compared using the independent samples t-test and, variables that were not normally distributed were compared with the Mann–Whitney U test. The Shapiro Wilk test was used for normality. Categorical variables were analyzed using the chi-squared test or Fisher’s exact test. Moreover, Kaplan–Meier analysis and the log-rank test were used to test for significant differences between the survival curves of the variables influencing the time to remission and to compare survival probabilities in order to identify significant clinical differences between the groups.

## 3. Results

### 3.1. Participants and Remissions

**Figure 3:**
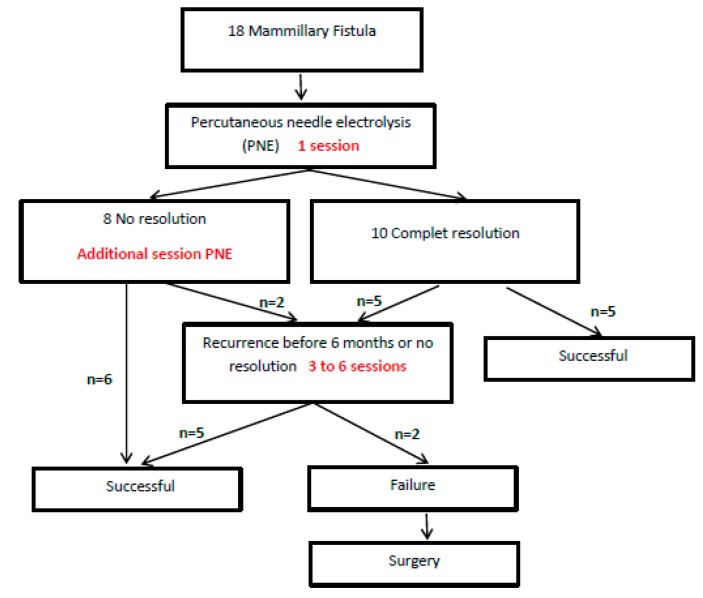
Flow diagram of the patients in this study.

The mean age ± SD of the 18 patients undergoing PNE was 47 ± 14.4 years (range: 18–67 years). Table 1 shows the characteristics of the patients in this study. Bacteriological examination was negative in six cases with breast abscess. This may be because the patients received antibiotic therapy before US-guided PNE. A mean ± SD of 2.56 ± 1.46 (range: 1–6) sessions of PNE were applied to each patient (Figure 3). The average time of the procedure was 8.3 ± 1.1 min (range: 7–10 min). Remission of the fistula without subsequent relapse occurred in 16 cases (89%) before exceeding the maximum of six sessions. The median number of sessions applied until remission was 2 (percentiles 25–75% = 3–2) and the median time until remission was three weeks (percentiles 25–75% = 8–2). No patients had complications associated with the application of the PNE during or after the session. During the application of the PNE, the median pain VAS score was 1/10 (percentiles 25–75 = 0–2). The mean time of the US-guided PNE sessions was 8.3 ± 1.1 min (range: 7–10 min). The mean length of follow-up was 16.7 ± 7.1 months (range: 8–24 months). 

### 3.2. Characteristics Influencing the Time to Remission (without Relapse within Six Months) 

Table 2 shows the hazard ratio and P-value of each characteristic estimated by the analyses using the Cox proportional hazard regression models. These univariate models indicate that the time to remission was more favorable in fistulas with an elongated appearance and show that the fistulas with an elongated appearance were 6.17 (95% CI: 1.33–28.55) times more likely to remit than those with an ovoid appearance. Moreover, a greater fistula width was seen to be associated with a lower likelihood of remission: for each 1 mm increase in width, the likelihood of remission is multiplied by 0.64 (95% CI: 0.47–0.88). Furthermore, ovoid fistulas present more abscesses than elongated fistulas; they require more sessions of PNE and the pain recorded after the treatment session was found to be greater.

**Figure 4:**
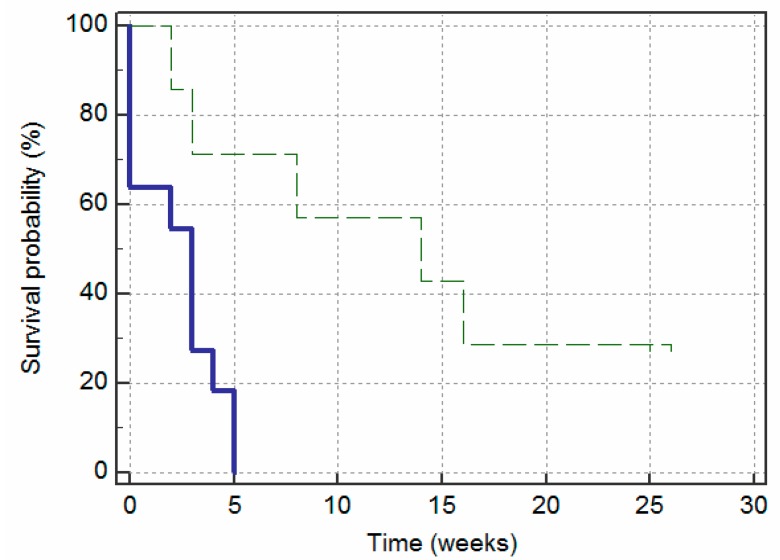
Kaplan–Meier survival curves demonstrating remissions over a period of 26 weeks, according to fistula appearance: elongated (solid line) and ovoid (dashed line).

The survival curves in Figure 4 reflect the effects of the appearance of the fistula—measured at baseline—on the likelihood of remission without relapse over the following six months. The graph shows that the fistulas with an elongated appearance had significantly less time without remission (i.e., survival time) than the fistulas with an ovoid appearance (X2 (1) = 8.34; *p* = 0.004). The cumulative survival probability of the elongated group decreased more rapidly over time (i.e., increased in the occurrence of remissions) than the ovoid group. The distinction between the two groups was evident from the first session, but it became most evident after the first three weeks. By looking at the Y-axis we can see that the 30th percentile of cumulative survival probability for the fistulas with an elongated appearance was three weeks and for those with an ovoid appearance, 15 weeks. The median of the probability of survival was 3 weeks and 14 weeks, respectively.

**Figure 5:**
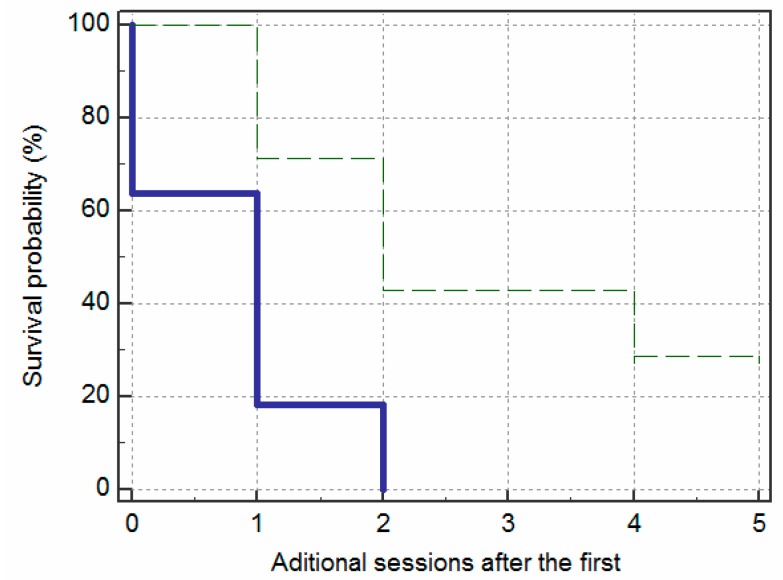
Kaplan–Meier survival curves demonstrating remissions over the number of additional PNE sessions, according to fistula appearance: elongated (solid line) and ovoid (dashed line).

**Figure 6:**
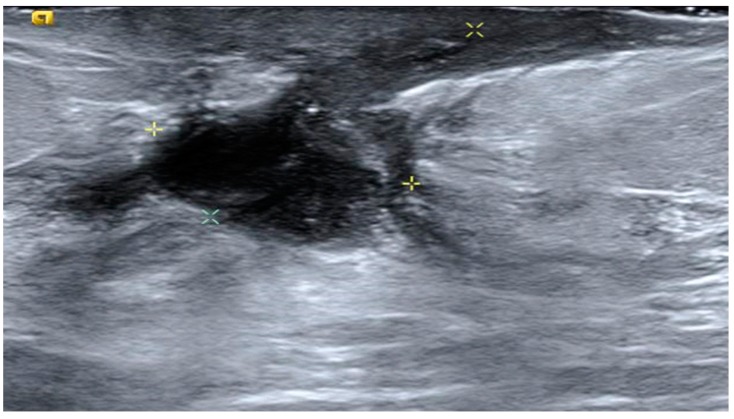
Example of an ovoid MF on ultrasound that was referred for surgery.

The survival curves in Figure 5 show that 2 sessions of PNE achieved remission in nine (81.8%) of the elongated fistulas, whereas the same number of sessions was only successful in two (28.6%) of the ovoid fistulas. Moreover, these two ovoid fistulas were characterized by not presenting an abscess. In contrast, the two elongated fistulas that needed up to three sessions were two fistulas without an abscess at the beginning. Failure of the PNE technique was observed in two cases with ovoid fistulas, which were referred for surgery (Figure 6).

## 4. Discussion

To our knowledge, this is the first study in which MFs have been managed using US-guided PNE. The findings obtained show that the procedure was successful in most cases. The management of MF is difficult. The incision and drainage technique is insufficient. Various surgical procedures for the treatment of MF have been reported [5,14,15,16,17]. The main problem is high recurrence [6,7].

US-guided PNE is an emerging minimally invasive approach, which involves the application of a galvanic current through an acupuncture needle acting as a negative electrode and inducing an electrochemical ablation in the area to which it is applied [10]. This technique provokes a local inflammatory process, allowing the phagocytes and affected tissue to repair [11]. It has been used successfully in various musculoskeletal pathologies, such as for the treatment of tendinopathies and epicondylitis [10,11,12]. An adapter was designed for this study to connect a 14G needle to the needle holder (cathode), enabling us to perform aspiration of the MF collections, irrigation with saline solution, and injection of an anesthetic inside the MF track to reduce the pain of the PNE technique. This cannot be done with the acupuncture needles usually employed, in the equipment used in the present study to perform PNE. Moreover, the same 14G needle inserted under US guidance into the MF track can be used for the whole procedure. Another important aspect of the PNE technique is its much localized (approximately 1 cm) ablation effect on the altered tissue. This is why we apply 5 impacts of 5 mA for 5 s at every 1 cm of the track, from the most distal part to the surface of the skin. PNE is more effective in elongated MFs because the width is usually <1 cm, while for ovoid MFs the width is ≥1 cm. Ultrasound examination was an excellent method for diagnosis, administration of therapy and follow-up.

Our experience has shown that MFs that reveal an elongated appearance on the US are generally resolved with two sessions of PNE, whereas ovoid MFs require several sessions of PNE for complete resolution and tend to recur. As for the MF appearance on US, it has also been reported that in patients with ovoid/rounded MFs the efficacy of an intralesional injection of corticoids is lower than in those with elongated MFs [18].

The findings should be interpreted in light of the methodological limitations of the study. Firstly, this is not a randomized study. Secondly, we used a small sample. This does not represent a major problem for the regression models conducted here. However, additional regression models adjusting abscess and appearance were not conducted and we cannot, therefore, identify if the presence of an abscess in ovoid fistulas is a relevant determinant. Otherwise, our subgroup analyses strongly show that abscess is not a determinant for elongated fistulas. Thirdly, the PNE protocol used in this study was based on the experience of the physiotherapists in our group. We believe further studies are necessary to optimize the PNE protocol, especially for the management of MFs with an ovoid appearance. However, the PNE treatment in this study was successful in most of the cases and the patients had no recurrence after a mean follow-up period of more than eight months. This finding is important because of the high rate of MF recurrence. Moreover, the patients tolerated the procedure very well and no complications were observed.

## 5. Conclusions

To the best of our knowledge, this is the first study in which the PNE technique has been used for the management of MF. The results obtained show that the technique is effective, simple, economical, quick, and well-tolerated. Most of the patients responded after two sessions of PNE. We recommend this procedure as an alternative to surgery.

## Figures and Tables

**Table 1:** Characteristics of all participants and by fistula appearance on ultrasound.

	All Samples (*n* * = 18)	Ovoid Appearance (*n* * = 7)	Elongated Appearance (*n* * = 11)	*p*-Value
Demographics and background				
Age (years), mean ± SD	41.4 ± 10.9	40.1 ± 5.8	42.3 ± 13.4	0.650
BMI, median (IQR **)	26.5 (5.0)	25.4 (3.3)	27.1 (6.0)	0.468
Smoker	14 (77.8)	6 (85.7)	8 (72.7)	0.518
With Diabetes	3 (16.6)	1 (14.2)	2 (18.1)	0.999
Fistula characteristics				
Duration of symptoms (months), median (IQR)	5.0 (8.0)	9.0 (41.0)	5.0 (9.0)	0.131
With inverted nipple	2 (11.1)	2 (28.6)	0 (0)	0.267
With fistulous orifice	12 (66.7)	6 (85.7)	6 (54.5)	0.199
With abscess	6 (33.3)	5 (71.4)	1 (9.1)	0.013
Location				0.205
Subareolar	3 (16.7)	2 (28.6)	1 (9.1)	
Periareolar	7 (38.9)	1 (14.3)	6 (54.5)	
Subareolar and periareolar	8 (44.4)	4 (57.1)	4 (36.4)	
Ultrasound image				0.066
Hypoechoic	9 (50.0)	2 (28.6)	7 (63.6)	
Anechoic	7 (38.9)	5 (71.4)	2 (18.2)	
Heterogeneous	2 (11.1)	0 (0)	2 (18.2)	
Width (mm), median (IQR)	5.5 (4.0)	9.0 (3.0)	5.0 (2.0)	0.003
Maximum diameter (mm), mean ± SD	16.7 ± 7.1	18.4 ± 6.2	15.6 ± 7.7	0.419
Response to treatment				
Pain (VAS) at the first session, median (IQR)	1.0 (2.0)	2.0 (2.0)	0 (1.0)	0.009

Data represent * *n* (%) unless otherwise noted. ** IQR = Interquartile range.

**Table 2:** Predictors of time to remission of the fistula (without relapse within six months) in Cox’s proportional hazards model.

Variables	Hazard Ratio (95% CI)	*p*-Value
Demographics		
Age (years)	1.00 (0.95–1.06)	0.955
BMI	1.11 (0.90–1.36)	0.336
Clinical characteristics		
Duration of symptoms (months)	0.98 (0.95–1.02)	0.313
Without inverted nipple	1.15 (0.26–5.15)	0.852
Without fistulous orifice	1.37 (0.48–3.89)	0.551
Non smokers	2.50 (0.70–8.95	0.159
Without Diabetes	3.58 (0.78–16.42)	0.100
Ultrasound characteristics (time-independent)		
Elongated appearance (vs. ovoid)	6.17 (1.33–28.55)	0.020
Without abscess	11.19 (1.40–89.36)	0.023
Location		
Subareolar	Ref.	
Periareolar	2.42 (0.47–12.40)	0.288
Subareolar and periareolar	1.40 (0.29–6.76)	0.798
Width(mm)	0.64 (0.47–0.88)	0.007
Maximum diameter (mm)	0.95 (0.87–1.04)	0.252
Variables	Hazard Ratio (95% CI)	
Demographics		
Age (years)	1.00 (0.95–1.06)	0.955
BMI	1.11 (0.90–1.36)	0.336
Clinical characteristics		
Duration of symptoms (months)	0.98 (0.95–1.02)	0.313
Without inverted nipple	1.15 (0.26–5.15)	0.852
Without fistulous orifice	1.37 (0.48–3.89)	0.551
Non smokers	2.50 (0.70–8.95)	0.159
Without Diabetes	3.58 (0.78–16.42)	0.100
Ultrasound characteristics (time-independent)		
Elongated appearance (vs. ovoid)	6.17 (1.33–28.55)	0.020
Location		
Subareolar	Ref.	
Periareolar	2.42 (0.47–12.40)	0.288
Subareolar and periareolar	1.40 (0.29–6.76)	0.798
Width(mm)	0.64 (0.47–0.88)	0.007
Maximum diameter (mm)	0.95 (0.87–1.04)	0.252
